# Hyperammonemic Encephalopathy: A Complication of Gastric Bypass Surgery

**DOI:** 10.7759/cureus.9864

**Published:** 2020-08-19

**Authors:** Varun Reddy, Jinal K Patel, Dilendra Weerasinghe, Johnathan Frunzi

**Affiliations:** 1 Internal Medicine, Medical Center of Trinity, Trinity , USA; 2 Internal Medicine, Medical Center of Trinity, Trinity, USA; 3 Surgery, Medical Center of Trinity, Trinity, USA

**Keywords:** hyperammonemia, roux-en-y gastric bypass, blood-bezoar, gastro-gastric fistula, hyperammonemia-encephalopathy, ammonia, astrocyte swelling

## Abstract

Hyperammonemia is a metabolic abnormality characterized by elevated levels of ammonia in the blood. This case report illustrates a 72-year-old Caucasian female with a history of prior gastric bypass surgery done 15 years ago, who was admitted multiple times for acute encephalopathy over the course of a few months. The patient was found to have a gastro-gastric fistula seen on a CT scan of the abdomen, which was the culprit of her acute encephalopathy. The patient underwent fistula closure via esophagogastroduodenoscopy.

## Introduction

Hyperammonemic encephalopathy is a condition that most often affects people suffering from liver cirrhosis. Approximately 4.5 million people in the US have liver cirrhosis, and approximately 30-45% of those may experience hyperammonemic symptoms such as lethargy, vomiting, confusion, irritability [1]. People with rare inborn genetic abnormalities, such as urea cycle deficiencies are also at increased risk for developing hyperammonemic encephalopathy compared to the general population [2].

Ammonia is a byproduct of amino acid degradation which occurs in the liver. Disruption of the various enzymatic processes in the liver can lead up to buildup of ammonia in the blood, thus leading to an encephalopathic state. Though the exact process is unclear, there are multiple theories as to how elevated levels of ammonia can affect the brain [1]. Regardless of the mechanism, autopsy findings have revealed that patients with hyperammonemia commonly show evidence of astrocyte swelling and neuronal toxicity [3].

There are multiple routes to control serum ammonia levels in individuals who are susceptible to hyperammonemia, one of which is limiting protein intake [2]. However, limiting protein intake is not viable in patients with liver cirrhosis as they commonly suffer from protein deficiencies and malnourishment. On the other hand, patients may be directed to therapies such as lactulose and rifaximin, which reduce ammonia reuptake in the gastrointestinal (GI) tract. In some cases, GI bleeds can provide the stimulus needed to trigger an encephalopathic episode, with digestion and reabsorption of the blood.

In more rare cases, hyperammonemic states can be seen in patients as a result of prior bariatric surgery. It is a rare complication, and most likely seen following Roux-en-Y gastric bypass surgery as opposed to other forms of bypass surgery [4,5]. Here we present a case report of an adult with gastro-gastric fistula secondary to gastric bypass surgery presenting as hyperammonemic encephalopathy.

## Case presentation

A 72-year-old Caucasian woman with a past medical history of atrial fibrillation, congestive heart failure, renal failure, nonalcoholic cirrhosis, and prior Roux-en-Y bypass surgery 15 years ago, presented to the emergency department for encephalopathy. The patient had been seen multiple times for similar episodes of encephalopathy over the course of eight months. However, during this particular episode, she was found unresponsive by friends in her home and was immediately brought to the hospital. On arrival, she was hemodynamically stable, but responsive only to deep painful stimuli. A stroke alert was called, and computed tomography (CT) of the brain was performed, which did not reveal acute changes. Initial laboratory investigation revealed a white blood cell count of 3.35 K/mcL, platelets of 135 K/mcL, potassium of 2.5 mmol/L, and serum ammonia of 58 umol/L. Urine analysis and chest x-ray were negative. The patient was monitored very closely and started on lactulose suppositories. Upon reexamination in the morning, she was alert, awake, oriented and her only complaint at that time was nausea and abdominal pain, which she stated had been present for several months.

**Figure 1:**
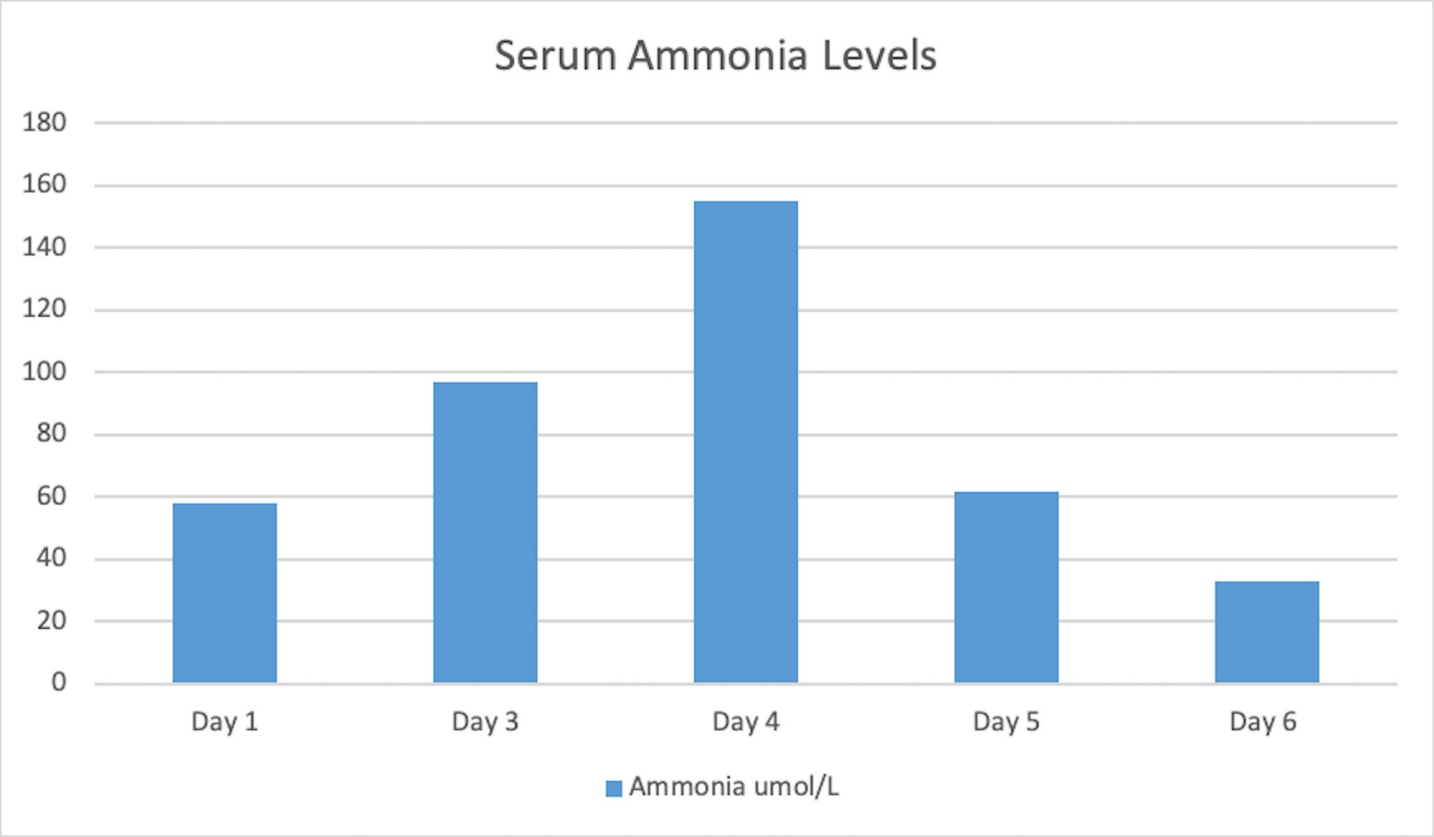
Serum Ammonia Levels Serum ammonia level through out the hospital course.

Repeat laboratory results revealed an increase in her serum ammonia levels of 97umol/L on day 3 (Figure 1). Family members had expressed concern that the patient had not been fully adherent to her medication regimen, knowing her history of gastric bypass surgery. Knowing this history, we began treatment for Wernicke’s encephalopathy with intravenous (IV) fluids, vitamins, replete electrolytes and continued to treat her hyperammonemia with lactulose and rifaximin.

**Figure 2:**
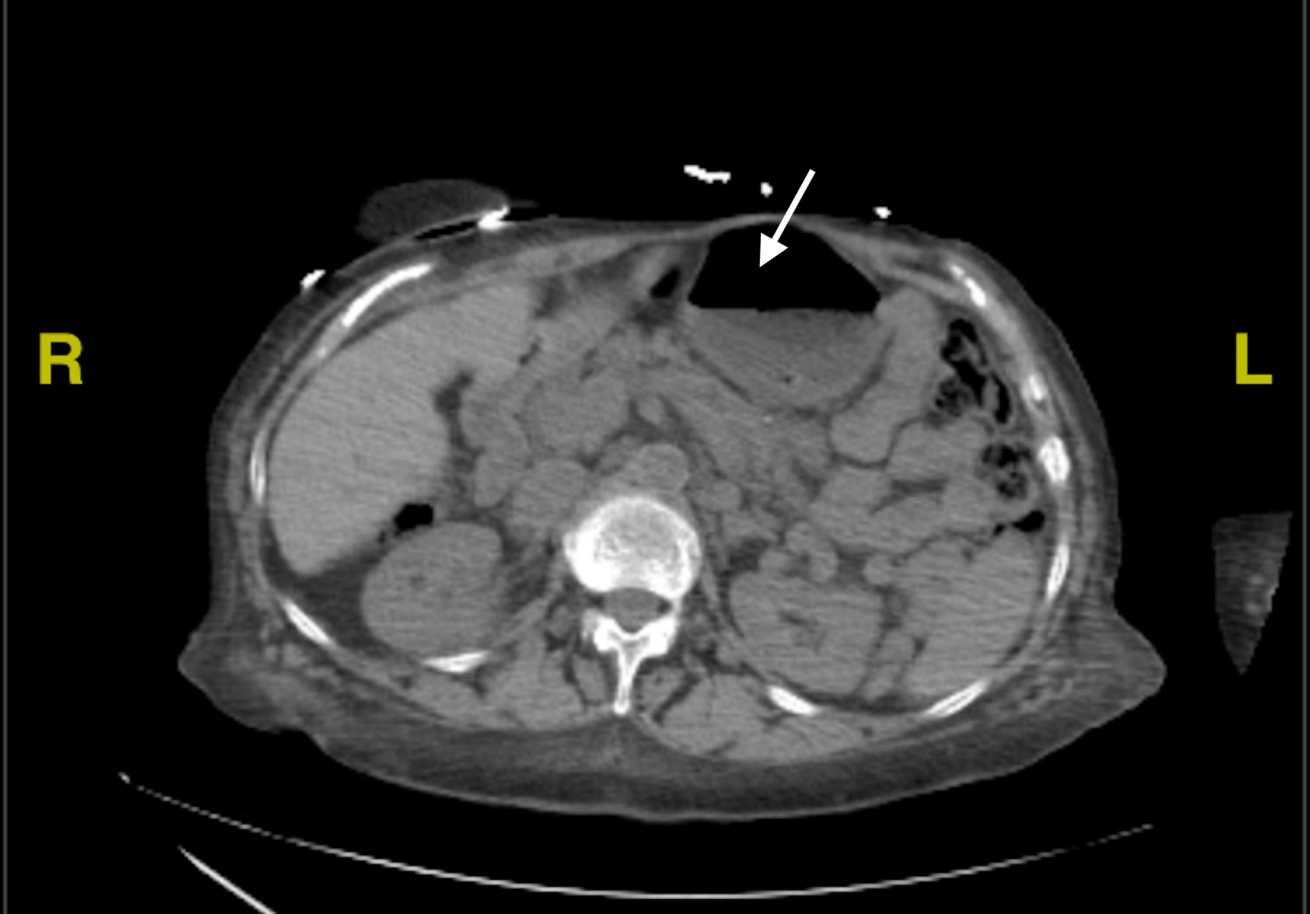
CT Abdomen and Pelvis showing gastric remnant with collection of blood Arrow shows Gastric remnant with collection of blood with in remnant

A bariatric surgeon was consulted as the patient had a history of gastric bypass surgery. Further review of prior CT scan of the abdomen and pelvis revealed dilation of the gastric remnant with air, indicating the presence of a gastro-gastric fistula (Figure 2). The CT scan also showed abnormalities in the remnant, which was suspected to be a collection of coagulated blood from an upper gastrointestinal bleed. An esophagogastroduodenoscopy (EGD) was performed and the fistula was closed via clips. Following the procedure, there was a sudden improvement in her nausea and abdominal pain. Her serum ammonia levels continued to fluctuate throughout the hospital stay (Figure 1) without any correlation to mental status changes.

## Discussion

In this case, we observed a patient with a history of nonalcoholic liver disease and prior gastric bypass surgery who presented with metabolic encephalopathy. She showed rapid improvement in mental status with seemingly no correlation with her serum ammonia levels. Further evaluation of the patient revealed a gastro-gastric fistula with a collection of coagulated blood in the remnant pouch. There are cases of toxic ingestion in which large amounts of rapidly ingested pills will form a drug bezoar in the stomach [6]. In such cases, the bezoar is continuously digested in the stomach, rendering medical management of the toxicity useless. Endoscopic removal of the bezoar is required for medical management to be effective. Similarly, in our patient, we saw a collection of blood in the gastric remnant. A gastro-gastric fistula had formed likely due to chronic inflammation secondary to marginal ulcers. Chronic bleeding into this blind pouch allowed a collection to form, similar to the bezoar in a drug overdose. As this remnant is separate from the rest of the GI tract, it is unaffected by medication like lactulose or rifaximin. In this isolated pouch, the blood-bezoar can be digested and reabsorbed at random, creating rapid fluctuations in serum ammonia levels.

Elevated serum ammonia levels can trigger encephalopathy, but changes in serum ammonia cannot be correlated with changes in mental status. Therefore, even though our patient showed improvement in her mental status despite an increase in serum ammonia, it is still likely that the slow digestion of a blood-bezoar in the gastric remnant acted as the stimulus for her encephalopathy. This is most likely the reason for the sudden increase in hospitalization for our patient, who had been doing well up until recent months. Closure of the fistula will allow for decompression of the gastric remnant and prevent further accumulation of blood. Patients with prior gastric bypass surgery are at risk for forming gastro-gastric fistula leading to increased risk of hepatic encephalopathy as described in our patient. Also, a potential other complication that can arise in the blind pouch is gastric varices which cannot be seen on diagnostic EGDs.

## Conclusions

This case adds to the limited body of data regarding gastric bypass complications. While gastro-gastric fistula itself is a well-known complication of gastric bypass, the resulting encephalopathy is a more dangerous sequel that can be easily missed. In this case, we highlight the relatively unknown but serious bypass related hyperammonemic encephalopathy. It is a condition that, if left unrecognized, has a high cost in the form of recurrent admissions and increased mortality. Physicians need to be aware of the potential outcomes in patients with a history of gastric bypass surgery and the need for potential early intervention to improve long term prognosis. 
